# Genetic Etiology of Neonatal Diabetes Mellitus in Vietnamese Infants and Characteristics of Those With *INS* Gene Mutations

**DOI:** 10.3389/fendo.2022.866573

**Published:** 2022-04-19

**Authors:** Can Thi Bich Ngoc, Vu Chi Dung, Elisa De Franco, Nguyen Ngoc Lan, Bui Phuong Thao, Nguyen Ngoc Khanh, Sarah E. Flanagan, Maria E. Craig, Nguyen Huy Hoang, Tran Minh Dien

**Affiliations:** ^1^ The Center of Endocrinology, Metabolism, Genetics, and Molecular Therapy, Vietnam National Children’s Hospital, Hanoi, Vietnam; ^2^ Pediatric Department, Hanoi Medical University, Hanoi, Vietnam; ^3^ Institute of Biomedical and Clinical Science, College of Medicine and Health, University of Exeter, Exeter, United Kingdom; ^4^ Institute of Genome Research, Vietnam Academy of Science and Technology (VAST), Hanoi, Vietnam; ^5^ Institute of Endocrinology and Diabetes, The Children’s Hospital at Westmead/Discipline of Child and Adolescent Health, University of Sydney, Sydney, NSW, Australia; ^6^ School of Clinical Medicine, University of New South Wales Medicine and Health, Discipline of Paediatrics and Child Health, Sydney, NSW, Australia

**Keywords:** diabetes mellitus in infants, INS mutations, neonatal diabetes mellitus, neonatal diabetes mellitus in Vietnamese infants, outcomes in infants with INS gene mutations

## Abstract

**Background:**

Neonatal diabetes mellitus (NDM) is a rare (1:90,000 newborns) but potentially devastating metabolic disorder characterized by hyperglycemia combined with low levels of insulin. Dominantly-acting insulin (*INS*) gene mutations cause permanent NDM through single amino acid changes in the protein sequence leading to protein misfolding, which is retained within the endoplasmic reticulum (ER), causing ER stress and β-cell apoptosis. Over 90 dominantly-acting *INS* gene mutations have been identified in individuals with permanent NDM.

**Patients and Methods:**

The study included 70 infants diagnosed with NDM in the first year of life between May 2008 and May 2021 at the Vietnam National Children’s Hospital. Sequencing analysis of all the genes known to cause NDM was performed at the Exeter Genomic Laboratory, UK. Clinical characteristics, molecular genetics, and annual data relating to glycemic control (HbA1c) and severe hypoglycemia of those with *INS* mutations were collected. The main outcomes of interest were HbA1c, daily insulin dose, growth, and cognitive/motor development.

**Results:**

Fifty-five of 70 infants (78.5%) with NDM harbored a mutation in a known disease-causing gene and of these, 10 had six different *de novo* heterozygous *INS* mutations. Mean gestational age was 38.1 ± 2.5 weeks and mean birth weight was 2.8 ± 0.5 g. They presented with NDM at 20 ± 17 weeks of age; 6/10 had diabetic ketoacidosis with pH 7.13 ± 0.26; plasma glucose level 32.6 ± 14.3 mmol/l and HbA1C 81 ± 15% mmol/mol. After 5.5 ± 4.8 years of insulin treatment, 9/10 have normal development with a developmental quotient of 80-100% and HbA1C 64 ± 7.3 mmol/mol, 9/10 have normal height, weight, and BMI on follow-up.

**Conclusions:**

We report a series of Vietnamese NDM cases with dominant *INS* mutations. *INS* mutations are the third commonest cause of permanent NDM. We recommend screening of the *INS* gene in all children diagnosed with diabetes in the first year of life.

## Introduction

Neonatal Diabetes mellitus (NDM) is defined by the presence of severe hyperglycemia associated with insufficient or no circulating insulin, occurring mainly before 6 months of age and rarely between 6 months and 1 year (1). The most frequent genetic causes of NDM resulting in abnormal β-cell function are methylation abnormalities of the chromosome 6q24 locus and mutations of the *ABCC8* or *KCNJ11* genes encoding the pancreatic β-cell potassium channel. Mutations in other NDM genes cause disease through a failure in pancreatic or β-cell development or as a result of the destruction of β-cells.

The human insulin gene (*INS*, OMIM # 176730) is located on the short arm of chromosome 11 (11p15.5) and encodes a single chain 110 amino acid peptide, preproinsulin, which is the insulin precursor (2, 3). Insulin biosynthesis begins by preproinsulin translocation from the cytoplasm into the endoplasmic reticulum (ER). Preproinsulin contains a signal peptide, B-chain, C-peptide, and A-chain (4). Preproinsulin is converted to proinsulin by removing the signal peptide. In proinsulin, the A chain links to B chain by C-peptide and proinsulin folds in the ER of the pancreatic β-cells. Only properly folded proinsulin molecules can transport to the Golgi apparatus and are converted to mature insulin by cleaving the C-peptide. The A-chain and B-chain of the mature insulin are crosslinked by two interchain disulfide bonds, namely, A7-B7, and A20-B19. The A-chain contains an internal disulfide bond between A6-A11.

Mutations in *INS* may affect the structure of preproinsulin, resulting in its abnormal processing to proinsulin or proinsulin misfolding (5). The abnormal or misfolded proinsulin gets retained in the ER, leading to severe ER stress and β-cell apoptosis. This process has been described in mouse models (6) and in humans (7, 8). Recent evidence suggests that *INS* mutations do not necessarily lead to β-cell death but rather the chronic ER stress interferes with β-cell growth and development (9). Mutations in *INS* cause permanent NDM (10), maturity-onset diabetes of the young (MODY10) (11), and hyperproinsulinemia (12). The first 10 heterozygous *INS* mutations causing NDM were reported in 2007 (13). Since then, over 50 dominantly-acting *INS* mutations have been reported. Autosomal recessive inheritance has also been described (14). In 2021, Støy et al. (15) reported 124 *INS* mutations in 389 diabetes cases referred to the Exeter Genomics laboratory, UK and suggested six mutation hotspots in *INS*, including A24, G32, F48, R89, C96 and c.188-31. The researchers also highlighted clinical heterogeneity in patients with mutations at these positions.

The phenotype of patients with diabetes due to a homozygous (or compound heterozygous) mutation in *INS* is characterized by severe intrauterine growth retardation (birth weight, <1 percentile) and neonatal diabetes, most likely reflecting severe insulin deficiency in the pre- and postnatal life, respectively. The majority of patients are diagnosed with diabetes in the first days or weeks of life and do not present with extrapancreatic manifestations (16). The phenotype usually resembles type 1 diabetes with requirement for insulin treatment. Insulin is a peptide hormone produced by beta cells of the pancreatic islets. It regulates the metabolism of carbohydrates, fats, and protein by promoting the absorption of glucose from the blood into the liver, fat, and skeletal muscle cells. In these tissues, the absorbed glucose is converted into either glycogen *via* glycogenesis or fats (triglycerides) *via* lipogenesis, or, in the case of the liver, into both. Insulin is secreted when blood sugar is high (as after a meal) and stops when blood sugar is low, and the liver releases glucose into the blood (15).

In our previous studies, we showed that dominant mutations in both subunits of the K_ATP_ channels resulted in a phenotype ranging from mild transient hyperglycemia to PND in our cohort. These children were diagnosed at the mean age of 8.7 ± 5.1 weeks and 51% had low birth weight (below 3rd percentile) (17). Hence, in this article, we report the mutation spectrum of NDM in individuals treated at the Vietnam National Children’s Hospital and focus on the outcomes in those with *INS* mutations.

## Research Design and Methods

### Individuals

The present study included 70 individuals who met the following criteria: (1) age at onset of diabetes <12 months; (2) hyperglycemia with fasting blood glucose ≥ 126 mg/dl (7.0 mmol/L) or random plasma glucose concentration ≥ 11.1 mmol/l and sustained for ≥ 2 weeks. Fasting was defined as no caloric intake for at least 4 h in children aged 0–1 years; (3) insulin dependence; and (4) exclusion of hyperglycemia caused by stress and infection and drug therapies.

### Molecular Genetic Analyses

Genomic DNA was extracted from peripheral blood using phenol/chloroform methods at Vietnam National Children’s Hospital. Mutation analysis was performed at the Exeter Genomic Laboratory, UK. Analysis of the coding regions and conserved splice sites of the *KCNJ11, ABCC8, INS, INSR, EIF2AK3, FOXP3, GATA4, GATA6, GCK, GLIS3, HNF1B, IER3IP1, PDX1, PTF1A, NEUROD1, NEUROG3, RFX6, SLC2A2, SLC19A2, WFS1*, and *ZFP57* genes was performed by a combination of Sanger sequencing and targeted next generation sequencing as previously described (18). Methylation–specific MLPA was performed to investigate methylation abnormalities on chromosome 6q24. Individuals without a mutation in a known NDM gene were considered for whole genome sequencing analysis to detect novel genetic causes.

The *INS* sequence was compared with the published reference sequence NM_000207.3 using Mutation Surveyor software version 2.61. When a novel variant was identified in the *INS* gene its frequency in population controls was assessed using the Genome Aggregation Database (gnomAD). The variants were crosschecked in variant databases, ClinVar, Leiden Open Variation Database (LOVD), and Human Gene Mutation Database (HGMD). The likely effect of the variant was predicted using the algorithms provided by VarSome tool (19). The variants were classified using the American College of Medical Genetics and Genomics (ACMG) best practice guidelines (20). Amino acid sequence of preproinsulin was adapted from Støy et al. (15). The three-dimensional structure 2KQP (21) of human proinsulin was used to visualize the predicted wild-type and mutant proinsulin using Swiss-PdbViewer (22). The intronic variant was analysed using the splice site prediction tools, Splice Site Prediction (https://www.fruitfly.org/seq_tools/splice.html) and Netgene2 (https://services.healthtech.dtu.dk/service.php?NetGene2-2.42).

### Clinical Phenotype and Biochemical Analyses

Clinical phenotype and biochemical tests of children with *INS* mutations were collected. The symptoms at onset and laboratory reports were obtained from medical records including sex, date of birth, gestational age, birth weight, age of diagnosis, and characteristics at diagnosis such as weight, height, presence of diabetic ketoacidosis (DKA), and neurological symptoms. Insulin and C-peptide were measured using immunoassay chemiluminescent technology by automated biochemistry Hitachi 704. Clinical follow-up started at 3 to 6 months intervals following diagnosis. Height and weight were measured in the last visit and BMI calculated, with SDS determined using WHO standards (23). The self-reported frequency of severe hypoglycemia was recorded.

HbA1c was measured at every visit using the automated Beckman Coulter AU2700/AU680 system. Blood glucose level and HbA1C targets were determined according to the International Society of Pediatrics and Adolescent Diabetes (ISPAD) 2018 guidelines (24). Serum ICA was determined by indirect immune-fluorescence and histochemical methods employing frozen unfixed human/primate or rodent pancreatic section as substrates. Autoantibodies to protein tyrosine phosphatase IA2, Zinc Transporter 8 (ZnT8A) were determined by enzyme immunoassay. GAD autoantibody was determined by an *in vitro* qualitative ELISA test.

This study was approved by the Ethics Committee of Vietnam National Children’s Hospital and all parents signed the informed consent.

### Statistical Analysis

Data analyses were performed using SPSS version 12.0 (SPSS Inc., Chicago, IL). Data are reported as frequency (%), mean ± standard deviation (SD), or median and range, as appropriate.

## Results

### Spectrum of NDM in Vietnam National Children’s Hospital

**Figure 1:**
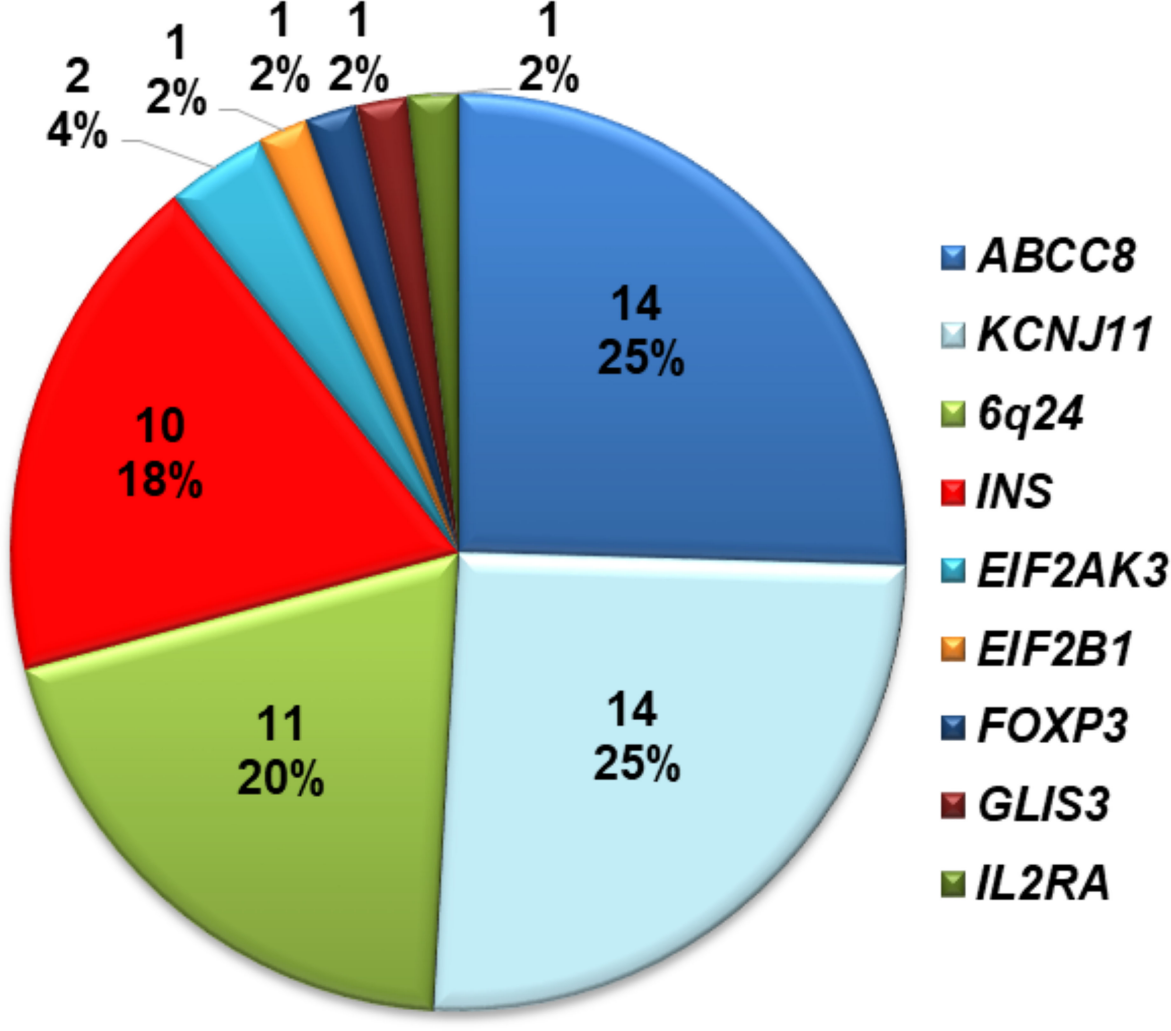
Distribution of mutations identified in 70 Vietnamese patients with diabetes diagnosed before 1 year of age.

Fifty-five of 70 infants (78.5%) with diabetes diagnosed before 12 months harbored a mutation in a known disease-causing gene. This including 54 individuals who were reported in our previous study (17) and one who was recently identified. Of these 28/55 (51%) had activating heterozygous *ABCC8* or *KCNJ11* mutations; 10 had heterozygous *INS* mutations; 11 had methylation abnormalities at chromosome 6q24; two had homozygous *EIF2AK3* mutations, one had a heterozygous *EIF2B1* mutation; one had a hemizygous *FOXP3* mutation; one had a compound heterozygous *GLIS3* mutation*;* and one had a compound heterozygous mutation in *IL2RA* (**Figure 1**). The ten infants with heterozygous *INS* mutations are described in detail in this study.

### Clinical Characteristics of Patients With *INS* Gene Mutations

Clinical characteristics are provided for all probands diagnosed under 1 year of age in whom an *INS* gene mutation was identified (**Table 1** and **Supplementary Table 1**). The mean age at diagnosis was 20 ± 17 weeks with 7/10 probands (70%) diagnosed with diabetes before 6 months of age (**Table 1** and **Supplementary Table 1**). Three of the 10 infants with *INS* mutations were diagnosed between 6 and 12 months. The presentation was either with symptomatic hyperglycemia (30%) or DKA (70%). The majority (9/10) were born appropriate for gestational age with the mean birth weight of 2.8 ± 0.6 kg corresponding from 10^th^ to 50^th^ percentile, only one case was small for gestational age with a corrected birth weight on the 8^th^ percentile (**Table 1**).

**Table 1:** Clinical characteristics of probands at diagnosis of diabetes resulting from an *INS* gene mutation.

	Age at diagnosis		All subjects
	0-6 months	> 6 – 12 months	
n	7	3	10
Sex (% male)	5 (71)	1 (33)	6 (60)
Birth weight (kg)	2.77 ± 0.68	3.0± 0.36	2.8 ± 0.59
Gestation age (week)	37.4± 2.7	39.6 ± 0.5	38.1± 2.5
Corrected birth weight (centile)	10-50	8-50	8-50
Age at diagnosis (months)	2.5± 2.0	9.7 ± 1.9	4.6 ± 3.9
C-peptide (nmol/l)	0.16 (0.036-1.09)	0.0003-0.8	0.4 ± 0.4
	Median (min-max)	Min - max	Mean ± SD
HbA1c (mmol/mol)	78.2 ± 45.4	89.3 ± 19.3	81.6 ± 38.6
Antibody status (Negative/Positive/NA)	4/0/3	1/0/2	5/0/5
DKA (%)	4 (57)	3 (100)	7 (70)

NA, not available; DKA, Diabetes Ketoacidosis.

By comparing the clinical features of children with an *INS* mutation with those with early-onset diabetes due to KATP channel mutations in our cohort we found that those with *INS* mutations presented with diabetes later (mean age 20 + 17 weeks vs 8.7 + 5.1, p=0.012) (**Table 2**).

**Table 2:** Comparison of clinical characteristics of children with diabetes caused by a mutation in *INS*, *KCNJ11*, or *ABCC8*.

	*INS*	*KCNJ11*	*ABCC8*	p*
n	10	14	14	
Age at diagnosis (weeks)	19.9 ± 17.0	8.5 ± 5.7	8.2 ± 5.0	0.015
Gestational age (weeks)	38.1± 2.5	38.8 ± 1.2	39.5 ± 1.1	
Birth weight (kg)	2.8 ± 0.59	2.7 ± 0.5	2.8 ± 0.3	
Birth weight < 3^rd^ (n)	0	7	7	
DKA	7	11	12	

*Differences between groups calculated using T- tests. DKA, Diabetes Ketoacidosis.

Five children with a heterozygous *INS* mutation were screened for β-cell auto-antibodies (anti-GAD, ICA, ZnT8); all five children were negative. The majority had no residual β-cell function as evidenced by low or undetectable basal or stimulated C-peptide levels.

### Molecular Genetic Analyses

Six *INS* mutations, p.L30V (c.88C>G), p.G32S (c.94G>A), p.C43S (c.127T>A), p.? (c.188-31G>A), p.R89C (c.265C>T), and p.C96R (c.286T>C) were identified in the 10 NDM patients (**Tables 3** and **4**). Of these, two mutations were each detected in three unrelated individuals [p.? (c.188-31G>A), and p.R89C (c.265C>T)], (**Table 4**). All variants were confirmed to have arisen *de novo* in the proband, were not presented in gnomAD and were classified as pathogenic variants in the ClinVar database (**Table 3**). All 5 missense variants were predicted to be deleterious by Mutation Taster, Sift, PolyPhen-2, Combined Annotation Dependent Depletion (CADD) and SNP & GO (**Supplementary Table 2**). The c.188-31>A mutation is located in intron 2 and predicted to create a new acceptor splice site with the introduction of 29 bp of intron 2 in the reading frame of exon 3 with a score of 0.62 and 0.44 in the Splice Site Prediction and Nextgene2, respectively (**Supplementary Figure 1**). According to the ACMG guidelines, all six *INS* variants were classified as pathogenic (**Table 3**).

**Table 3:** *INS* mutations identified in Vietnamese patients with neonatal diabetes mellitus.

Location	cDNA change (NM_000207.3)	Amino acid change (NP_000198.1)	Effect	LOVD	ClinVar	SNP id	HGMD	ACMG classification	Reference
Exon 2	c.88C>G	L30V	Missense	0000786601	–	–	CM081668	Pathogenic (PS2, PM1, PM2, PM5, PP3, and PP5)	(7)
Exon 2	c.94G>A	G32S	Missense	0000786583	VCV000021122 Pathogenic	rs80356664	CM074280	Pathogenic (PS1, PS2, PM1, PM2, PM5, PP3, and PP5)	(13, 25)
Exon 2	c.127T>A	C43S	Missense	–	–	–	CM154067	Pathogenic (PS2, PM1, PM2, PM5, and PP3)	(15)
Intron 2	c.188-31G>A	p.?	Splicing	0000473813	VCV000211186 pathogenic	rs797045623	CS120217	Pathogenic (PS2, PS3, PM2, PM4, PP3, and PP5)	(26)
Exon 3	c.265C>T	R89C	Missense	0000786591	VCV000021117 pathogenic	rs80356669	CM074283	Pathogenic (PS2, PM1, PM2, PM5, PP3, and PP5)	(13)
Exon 3	c.286T>C	C96R	Missense	–	VCV000918067 Pathogenic	rs1845839718	CM128900	Pathogenic (PS1, PS2, PM1, PM2, PM5, PP3, and PP5)	(13)

LOVD, Leiden Open Variation Database; HGMD, Human Genetic Mutation Database; ACMG, American College of Medical Genetics and Genomics.

**Table 4:** Clinical and biochemical characteristics at last evaluation.

Patient	Current age (years)	Insulin requirement dose U/kg/day	HbA1c (mmol/mol)	Height at last evaluation cm/(SDS)	BMI (kg/m^2^)/(SDS)	*INS* mutations
1	17	0.78	67	151.5 (-3.0)	16.7 (-2.1)	c.127T>A (p.C43S)
2	2.8	1.02	63	124 (-1.98)	14.4(-1.2)	c.188-31G>A (p.)?
3	8.1	0.56	65	117 (-1.94)	14.3 (-1.1)	c.188-31G>A (p.)?
4	8.5	0.77	67	135 (0.95)	16.7 (0.3)	c.286T>C (p.C96R)
5	6.8	1.1	62	123 (0.68)	15.2 (-0.07)	c.265C>T (p.R89C)
6	2.8	0.45	56	93.3 (-0.38)	16.0 (-0.03)	c.265C>T (p.R89C)
7	4.1	0.55	69	104 (-0.09)	13.4 (-2.4)	c.94G>A (p.G32S)
8	1.3	0.67	67	82 (0.66)	15.3	c.188‐31G>A (p.)?
9	2.5	0.56	79	89.5 (-0.47)	15.3 (-0.82)	c.88C>G (p.L30V)
10	1.3	0.2	52	78 (0.18)	14.7	c.265C>T (p.R89C)
X ± SD	5.5 ± 4.8	0.6 ± 0.2	64.7± 7.3			

**Figure 2:**
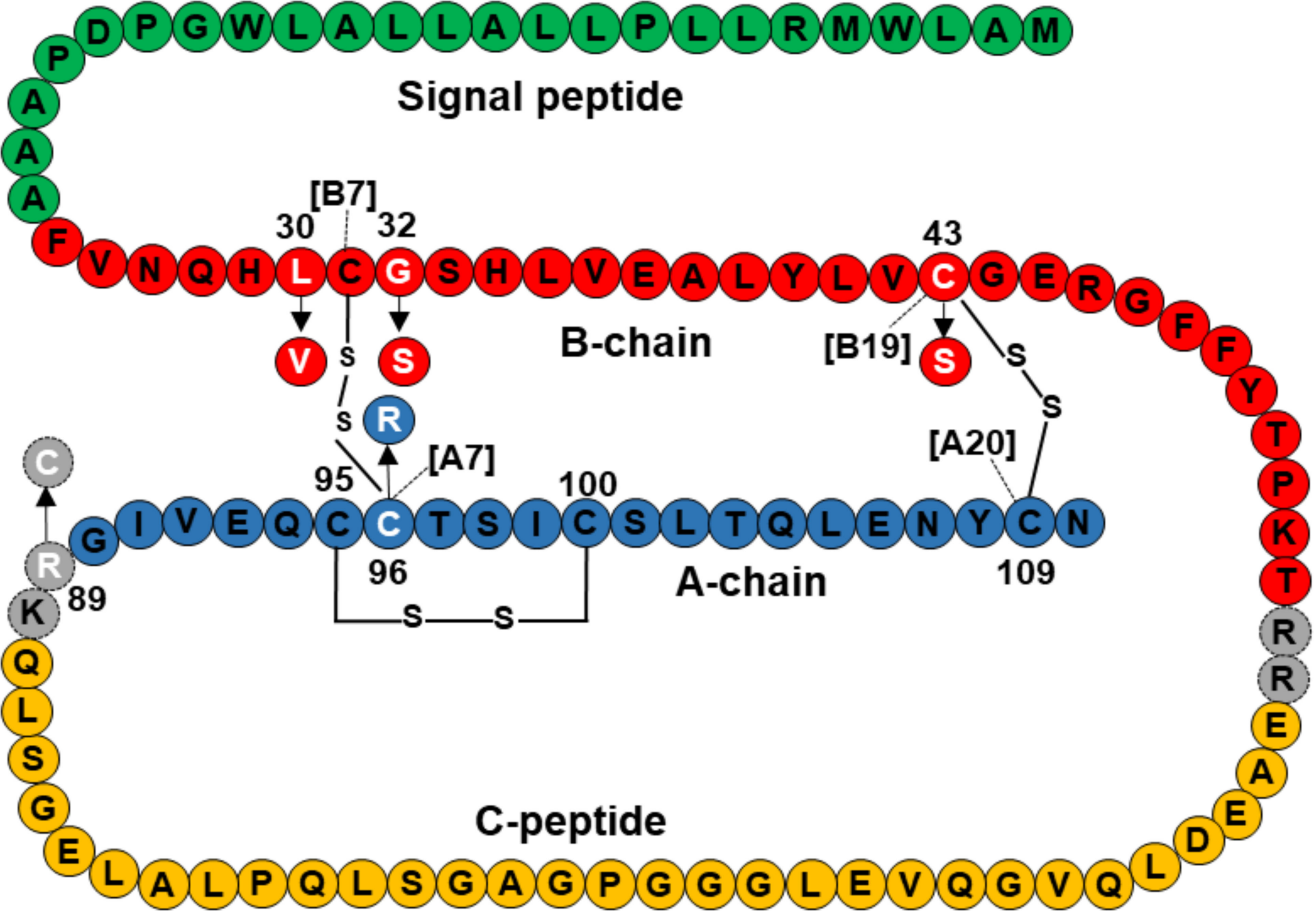
Location of *INS* mutations identified in 10 Vietnamese patients in the preproinsulin. Green, red, orange, and blue color amino acids represent for signal peptide, B-chain, C-peptide, and A-chain, respectively. Mutations are marked in white, including L30V, G32S, C43S, R89C, and C96R. Amino acid sequence of preproinsulin was adapted from Støy et al. (15).

Three mutations, p.L30V, p. G32S, and p.C43S are located in the B-chain of preproinsulin, and p.C96R is located in the A-chain of preproinsulin (**Figure 2**). The mature insulin includes three disulfide bonds, including A6-A11 (corresponding to C95-C100 in preproinsulin chain), B7-A7 (corresponding to C31-C96 in preproinsulin chain), and B19-A20 (corresponding to C43-C109 in preproinsulin chain). Therefore, the two mutations p.C43S and p.C96R are predicted to result in a loss of disulfide bonds C43-C109 and C31-C96, respectively (**Supplementary Figure 2A, B**). In addition, a change to arginine at codon 96 might be forming clash bonds of R96 with C31 (**Supplementary Figure 2B**).

### Treatment

All children with an *INS* mutation were treated with insulin. They have been followed up for 5.5 ± 4.8 years. The mean HbA1c at the last visit was 64.7 ± 7.3 mmol/mol (**Table 4**). Except for case 1, the height, weight and BMI were normal on follow-up. We screened for but did not detect chronic complications such as: retinopathy, albuminuria in cases 1, 3, 4, 5. Neurodevelopment was normal in all cases.

There were no episodes of severe hypoglycemia, defined as losing consciousness or having seizures (22), reported over the course of the follow-up in all patients (18).

## Discussion

In our comprehensive mutation analysis of a large cohort of 70 infants with diabetes diagnosed before 12 months of age enrolled at Vietnam National Children’s Hospital, we identified gene mutations in 55 infants (78.5%) as previously described (17). The mutation pick-up rate in our study was similar to that in Slovakian infants (27), but lower than that in Ukrainian (28) and Chinese infants (29). In our cohort, mutations in the *INS* gene were the third most common genetic etiology after KATP channel mutations and chromosome 6q24 methylation abnormalities (**Figure 1**). This difference may be due to ethnicity, race, or study sample size.

Eight of the ten individuals had pathogenic variants located in a mutation hotspot highlighted by Støy et al. (15) namely p.G32S, p.R89C (n=3), p.C96R, and c.188-31G>A (n=3). Three of these, p.G32S, p.R89C, and p.C96R have been commonly reported across different ethnic groups (30–32).

Mutation at the B19 (C43) has been shown to result in completely retained of the *INS* protein in ER in *in vitro* experiments (33). Therefore, C43S disrupts the B19-A20 disulfide bond, leading to proinsulin accumulation which causes ER stress. The B7-A7 (C31-C96) is the second disulfide bond to create proinsulin folding. In our study, only patient 4 carried the p.C96R mutation. Balboa et al. (9) generated induced pluripotent stem cells (iPSCs) from a Finnish patient harboring the *INS* p.C96R mutation and CRIPSPR/SpCas9 corrected the iPSC lines, and differentiated them into β-like cells *in vitro*. The results showed that the mutation enhanced ER-stress and reduced proliferation in *INS*-mutant β-like cells without increasing apoptosis. Therefore, p.C96R leads to a defect of β-cell mass expansion, which is associated with diabetes development. Another mutation affecting C96, p.C96Y, has been studied in Akita mice (carrying the insulin 2 *Ins2* ^+/C96Y^ mutation) to improve understanding of this mutation in diabetes pathogenesis, for example, misfolded proinsulin, ER stress, β-cell apoptosis or proliferation defect, or diabetes phenotype heterogeneity between male and female Akita mice (34–39).

The residue L30 (B6) could be involved in orienting the N-terminal region, maintaining local structure in the vicinity of the B7-A7 disulfide, or making contact with a receptor (40). The modeling of insulin also indicated L30 (B6) interacts with C95 (A6) *via* a hydrogen bond (distance = 2.06 Å) and the mutation p.L30V increases B6-A6 distance to 2.74 Å (**Supplementary Figure 3**). p.L30M and p.L30P increase Cys-Cys distance between B7-A7 to 7.53 and 5.98 Å, respectively, compared to 2.27 Å of wild-type L30 (8). Therefore, p.L30M and p.L30P disfavor disulfide bond formation and change the structure after disulfide bond formation. Functional assay showed that p.L30M and p.L30P perturbed proinsulin structure and lead to defective insulin secretion. The mutation p.L30V may act in the similar way to p.L30M and p.L30P, resulting a defect in proinsulin folding. The mutation p.L30V was first reported in a child with NDM (7) who showed a later onset (184 day-old versus 23 day-old) and a higher insulin dose (0.85 versus 0.56 U/kg/day) at the age of 30 months, compared to our case 9 (**Supplementary Table 1** and **Table 4**).

Støy et al. (13) suggested that G32 (B8) plays an important structural role by participating in a β-turn adjoining the central α-helix that lies in the proximity to the B7-A7 (C31-C96) disulfide bond. The substitution of L-serine for glycine at B8 (p.G32S) reorients the B8 conformation, resulting in rotation of B1-B8, turning the B7 cysteine away from its partner at A7. The p.G32S was proved to partially retain the protein in the ER and recruit to granules, and decrease co-transfected wild-type secretion (33). Such results explain the mechanism through which p.G32S causes diabetes.

A previous study investigating the mRNA of an individual heterozygous for the c.188-31G>A mutation detected aberrant transcripts with an insertion of 29 bp of intron 2 to exon 3 (26). This splice site mutation has been observed in the Caucasian population in Spain (26), United States (41), Czech Republic (42), and Japan (43) as causing NDM or maturity-onset diabetes of the young. Our study is the second report of this mutation in Asian individuals. Panova et al. (44) have successfully generated a neonatal diabetes-specific iPSC line harboring c.188-31G>A which may be used to investigate how the mutation affects insulin accumulation in the β- cells.

The p.R89C mutation creates an additional unpaired cysteine, which may disrupt disulfide bond formation in proinsulin. p.R89C is located at a CpG dinucleotide, a hotspot for pathogenic mutations in the human genome. This mutation hinders normal folding, leading to a reduction of properly folded protein. Rajan et al. (33) showed that p.R89C resulted in aberrant processing of proinsulin to insulin, however, no significant depletion of wild-type insulin secretion was observed.

In our study, seven children with *INS* gene mutations had diabetes onset before 6 months of age whilst three were diagnosed between 6 months and 12 months. The median age at diagnosis of the *INS* gene mutation carriers was 20 ± 17 weeks later than in Edghill’s study (25), in which heterozygous *INS* mutations were found in 33/141 (23%) probands diagnosed before 6 months, 2/86 (2%) between 6 and 12 months, and none of 58 diagnosed between 12–24 months of age. In our cohort, 3 with *INS* mutations were diagnosed after the age of 6 months. Taken together, these data suggest that *INS* mutations are more common in infants presenting with NDM at a younger age, however further large cohort studies are needed to establish the prevalence of *INS* mutations in patients presenting at a range of ages, especially in those who do not have autoantibodies to islet cell proteins. A recent paper by Støy et al. reported that in a cohort of 274 individuals with *INS* dominant mutations, 65% were diagnosed before 6 months of age and 18% of patients between 6 and 12 months of age. Thus by testing individuals diagnosed with diabetes before 12 months for the *INS* gene, one would expect to pick up >80% of cases caused by pathogenic dominant variants in this gene (15) We therefore recommend that the *INS* gene should be screened in all children diagnosed with diabetes in the first year of life and not just before the age of 6 months, as well as those at an older age with features of monogenic diabetes including absent autoantibodies, in keeping with the ISPAD guidelines (45).

The majority of other NDM genetic subtypes result in intrauterine growth restriction (IUGR) or low birth weight, described as small for gestational age (SGA), which is due to insulin deficiency *in utero* (46). This is however not the case for dominant *INS* mutations as confirmed in our cohort where the mean birth weight was normal (2.8 ± 0.5kg, range 1.5–3.6) adjusted for gestational age, corresponding from the 8th percentile to 50^th^ percentile.

Management of diabetes in infants and adolescents is a challenge. According to the ISPAD guidelines (24), the target HbA1c is < 7.0% for children, adolescents and young adults who have access to comprehensive care. In our study, only one case achieved the target HbA1c. While our cohort is small, this suggests that achieving the target glucose control in this population is challenging. Use of continuous glucose monitoring and insulin pump therapy may improve glycemic control, but universal funding for these technologies is not currently available in Vietnam.

Neurological dysfunction is a key feature of the phenotype of some patients with PNDM due to mutations in *KCNJ11* or *ABCC8* (47) but patients with *INS* gene mutations in our study did not have other associated extra-pancreatic features, including neurological dysfunction. This is consistent with previous studies (16).

A limitation of our study is the small sample size; however our data add to the limited published longitudinal data on children with *INS* mutations. It will be important to follow this cohort further to determine their risk of vascular complications compared with other forms of NDM and type 1 diabetes.

## Conclusion

We report and characterise a series of children with *INS* gene mutations. These mutations are the third most common cause of diabetes in the first year of life. We recommend that the *INS* gene should be screened for mutations in all children diagnosed with diabetes before 12 months of age who are antibody negative. Long term follow-up of these children is important to inform clinical care and monitoring for complications.

## Data Availability Statement

The datasets presented in this study can be found in online repositories. The names of the repository/repositories and accession number(s) can be found below: All disease-causing variants identified by next generation sequencing in this project were uploaded onto the DECIPHER database (https://decipher.sanger.ac.uk/).

## Ethics Statement

The studies involving human participants were reviewed and approved by Vietnam National Children’s Hospital IRB#1. Written informed consent to participate in this study was provided by the participants’ legal guardian/next of kin.

## Author Contributions

CN and VD conceptualized, designed the study, and wrote and reviewed the manuscript. CN, VD, BT, and NK provided patients’ clinical information. MC reviewed/edited the manuscript. NL, EF, SF, TD, NH, and MC analyzed data, and wrote and reviewed the manuscript. All authors contributed to the article and approved the submitted version.

## Funding

EF is a Diabetes UK RD Lawrence fellow. Testing for neonatal diabetes was funded by a Wellcome Trust Senior Investigator Award to Sian Ellard and Andrew Hattersley.

## Conflict of Interest

The authors declare that the research was conducted in the absence of any commercial or financial relationships that could be construed as a potential conflict of interest.​​​​​​​​​​​​​​​​​​​​

The reviewer NS declared a past co-authorship with one of the authors EF to the handling editor.

## Publisher’s Note

All claims expressed in this article are solely those of the authors and do not necessarily represent those of their affiliated organizations, or those of the publisher, the editors and the reviewers. Any product that may be evaluated in this article, or claim that may be made by its manufacturer, is not guaranteed or endorsed by the publisher.
