# Supplementary material for: Genetic Etiology of Neonatal Diabetes Mellitus in Vietnamese Infants and Characteristics of Those With INS Gene Mutations
Source: Front Endocrinol (Lausanne). 2022 Apr 19;13:866573. doi: 10.3389/fendo.2022.866573 (PMC9063464; doi:10.3389/fendo.2022.866573)
Supplement: Supplementary file 1 [file DataSheet_1.docx]

**Supplementary Table 1.** Clinical, biochemical, and molecular characteristics of the infants at diagnosis

| **Patient** | **GA (week)** | **BW (kg)** | **BW centile** | **Age (week)** | **Glucose (mmol/l)** | **HbA1c mmol/mol** | **Urinary ketone** | **C-peptide** | **Antibody** | **Seizure** | **DKA** | ***INS* mutations** |
| --- | --- | --- | --- | --- | --- | --- | --- | --- | --- | --- | --- | --- |
| 1 | 40 | 3.6 | 50 | 25.7 | 24 | 67 | + | NA | NA | - | Severe | c.127T>A (p.C43S) |
| 2 | 39 | 3.2 | 40 | 19 | 34.7 | 116 | + | NA | NA | - | Severe | c.188-31G>A |
| 3 | 37 | 2.5 | 10 | 10 | 54 | 70 | + | 0.16 | - | - | Severe | c.188-31G>A |
| 4 | 40 | 3.4 | 50 | 51 | 21 | 101 | + | 0.0 | - | - | Severe | c.286T>C (p.C96R) |
| 5 | 32 | 1.5 | 10 | 3 | 44.4 | 19 | - | 0.15 | - | - | No | c.265C>T (p.R89C) |
| 6 | 36 | 2.7 | 50 | 2 | 27.8 | 125 | + | 0.036 | NA | - | Severe | c.265C>T (p.R89C) |
| 7 | 39 | 2.9 | 10 | 34.8 | 18.1 | 102 | - | 0.56 | NA | - | No | c.94G>A (p.G32S) |
| 8 | 39 | 3.2 | 50 | 11.7 | 19.3 | 103 | - | 0.35 | - | - | No | c.188‐31G>A |
| 9 | 39 | 2.7 | 10 | 3.2 | 16.65 | 14 | + | 1.09 | - | - | Mild | c.88C>G (p.L30V) |
| 10 | 40 | 2.7 | 8 | 38.5 | 52.2 | 99 | + | 0.8 | - | - | Severe | c.265C>T (p.R89C) |
| X±SD |  | 2.8±0.5 |  | 20±17 |  |  |  |  |  |  |  |  |

NA, not available; “+”, positive; “-”, negative; GA, Gestational age; BW, Birth weight; DKA, Diabetes Ketoacidosis

**Supplementary Table 2.** In silico predicted effects of five missense mutations in *INS* identified in Vietnamese patients with NDM

| **Location in *INS*** | **cDNA change (NM_000207.3)** | **Amino acid change**  **(NP_000198.1)** | **Coordinates** | **SIFT** | **PolyPhen2** | **CADD** | **Mutation Taster** | **SNPs&GO** | |
| --- | --- | --- | --- | --- | --- | --- | --- | --- | --- |
| Exon 2 | c.88C>G | L30V | chr11:2182114G>C | Deleterious  (0) | Damaging (1.0) | Likely deleterious (24.0) | Disease causing (1) | Disease  (0.808) |  |
| Exon 2 | c.94G>A | G32S | chr11:2182108C>T | Deleterious  (0) | Damaging (1.0) | Likely deleterious (26.2) | Disease causing (1) | Disease  (0.895) | |
| Exon 2 | c.127T>A | C43S | chr11:2182075A>T | Deleterious  (0) | Damaging (1.0) | Likely deleterious (24.8) | Disease causing (1) | Disease  (0.947) |  |
| Exon 3 | c.265C>T | R89C | chr11:2181150G>A | Deleterious  (0) | Damaging (1.0) | Likely deleterious (24.1) | Disease causing (1) | Disease  (0.768) |  |
| Exon 3 | c.286T>C | C96R | chr11:2181129A>G | Deleterious  (0) | Damaging (1.0) | Likely deleterious (25.4) | Disease causing (1) | Disease  (0.922) |  |

Numbers in blanket in the PolyPhen2, Mutation Taster, and SNPs&GO indicates disease probability of the mutations (If probability > 0.5, the mutation is predicted disease). Numbers in blanket in the SIFT and CADD represent scores of the mutations. Mutations with SIFT scores in the range 0.0 to 0.05 are considered deleterious.


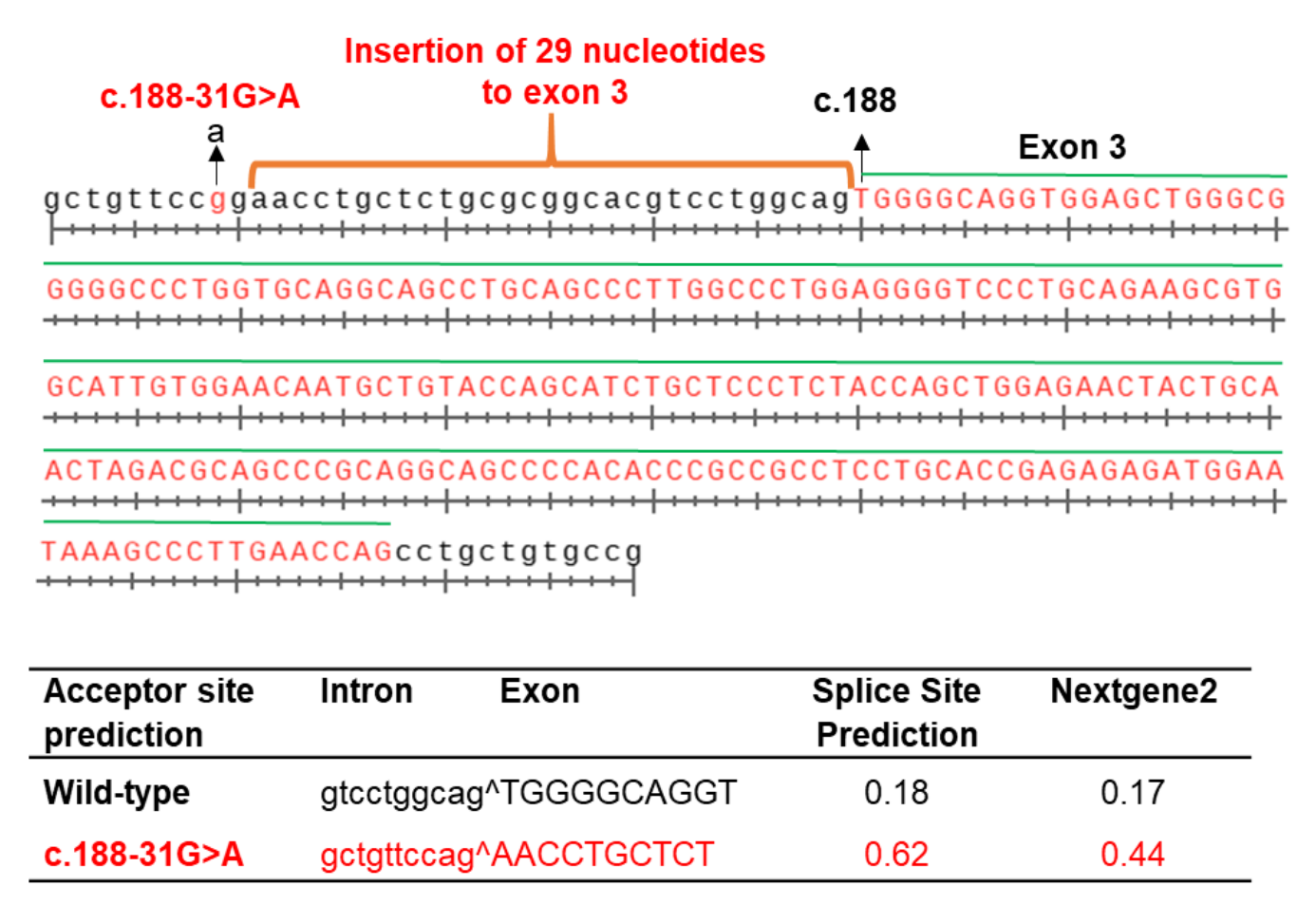


**Supplementary Figure 1.** Mutation c.188-31G>A in intron 2 of *INS***.** Upper part: location of mutation in *INS* gene and 29 nucleotides may involve in exon 3. Lower part: *in silico* analysis of mutation c.188-31G>A with Splice Site Prediction (https://www.fruitfly.org/seq_tools/splice.html) and Netgene2 (https://services.healthtech.dtu.dk/service.php?NetGene2-2.42).


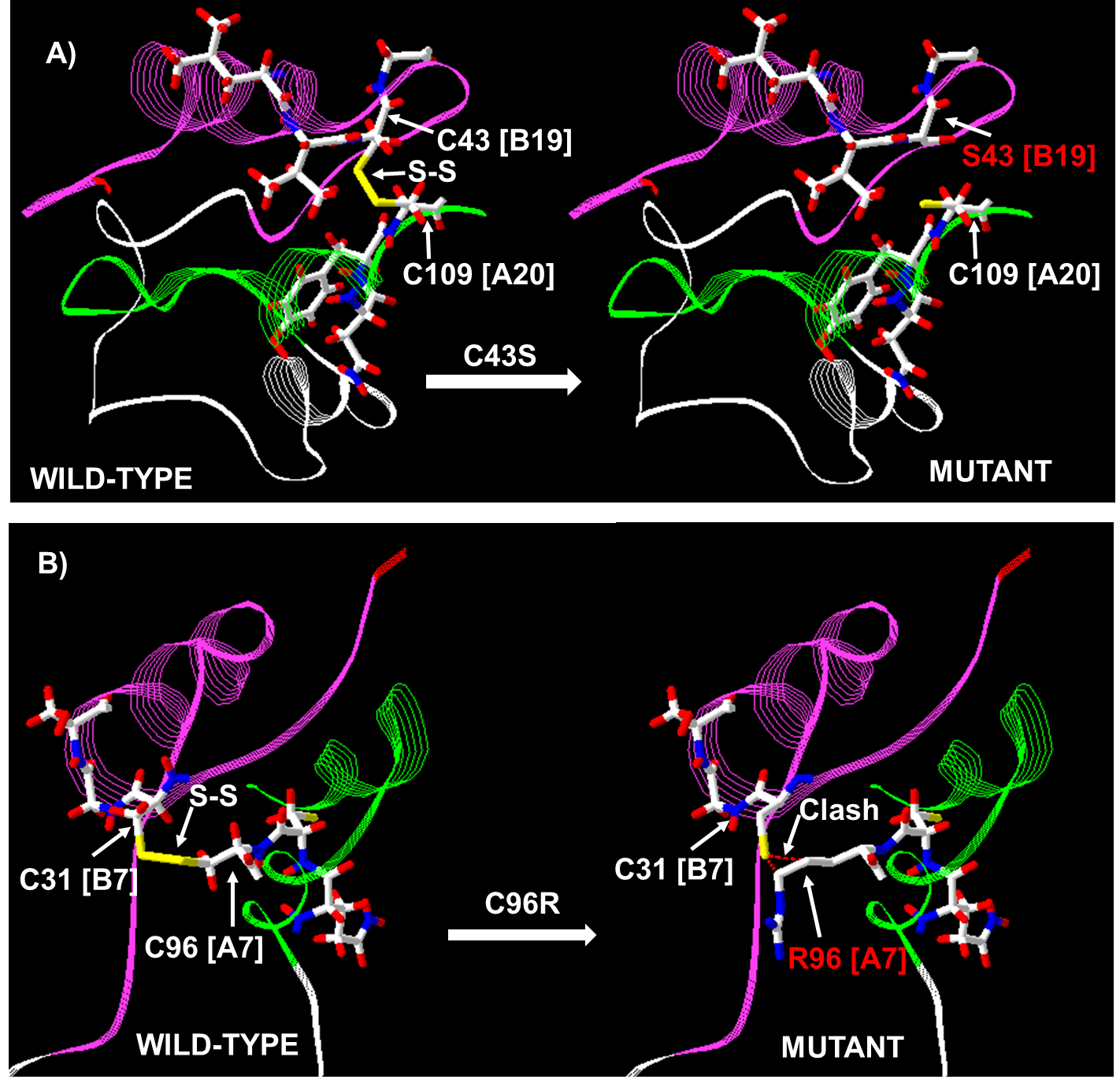


**Supplementary Figure 2.** Mutations C43S and C96R in three-dimensional structure of proinsulin (PDB ID: 2KQP). A) Mutation C43S cause a loss of disulfide bond between B19-A20 (C43-C109). B) Mutation C96R disrupts disulfide bond between B7-A7 (C31-C96). Disulfides bonds are marked in yellow. Mutation S43 and R96 are marked in red.

**
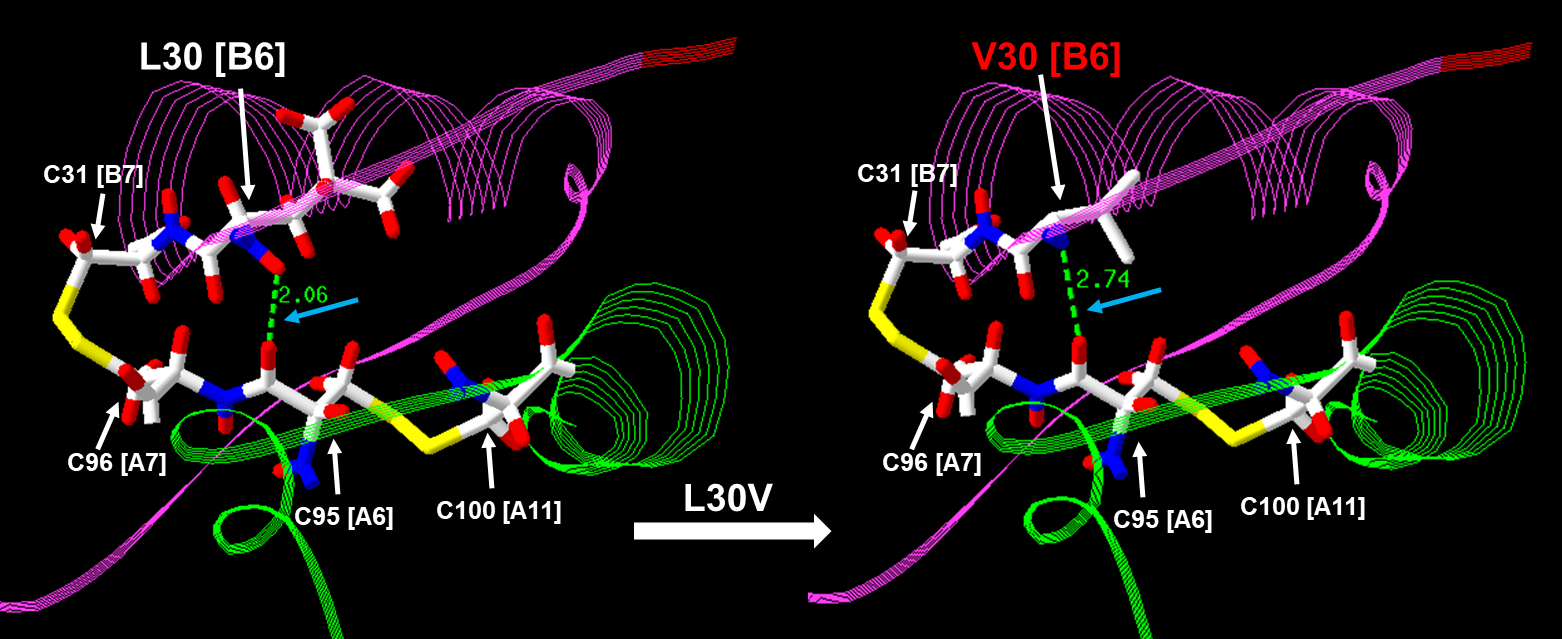
**

**Supplementary Figure 3.** Three-dimensional structure of L30V in proinsulin (PDB ID: 2KQP). Mutation V30 is marked in red. Mutation L30V increases distance between B6 and A6 to 2.74 Å, compared to 2.06 Å of wild-type L30.
